# Mitoepigenetics and gliomas: epigenetic alterations to mitochondrial DNA and nuclear DNA alter mtDNA expression and contribute to glioma pathogenicity

**DOI:** 10.3389/fneur.2023.1154753

**Published:** 2023-05-30

**Authors:** Clare I. Grady, Lisa M. Walsh, John D. Heiss

**Affiliations:** ^1^Neurosurgery, MedStar Georgetown University Hospital, Washington, DC, United States; ^2^Surgical Neurology Branch, National Institute of Neurological Disorders and Stroke (NINDS), National Institutes of Health, Bethesda, MD, United States

**Keywords:** mitoepigenetics, epigenetics, glioma, mitochondria, glioblastoma, mtDNA, methylation, noncoding RNA

## Abstract

Epigenetic mechanisms allow cells to fine-tune gene expression in response to environmental stimuli. For decades, it has been known that mitochondria have genetic material. Still, only recently have studies shown that epigenetic factors regulate mitochondrial DNA (mtDNA) gene expression. Mitochondria regulate cellular proliferation, apoptosis, and energy metabolism, all critical areas of dysfunction in gliomas. Methylation of mtDNA, alterations in mtDNA packaging via mitochondrial transcription factor A (TFAM), and regulation of mtDNA transcription via the micro-RNAs (mir 23-b) and long noncoding RNAs [RNA mitochondrial RNA processing (RMRP)] have all been identified as contributing to glioma pathogenicity. Developing new interventions interfering with these pathways may improve glioma therapy.

## 1. Introduction

Adult diffuse gliomas are Central nervous system (CNS) tumors arising from glial cells, most often astrocytes, oligodendrocytes, and ependymal cells. In 2016, the World Health Organization (WHO) published the fourth version of the CNS tumor classification system with the underlying concept of a multiple-input or layered diagnostic design based on histology, grading, and genomic markers (1). This new, layered classification of diffuse gliomas is better suited to clinical practice than earlier ones because it better predicts prognosis and the choice of therapies for biologically and genetically similar tumors. Glioblastoma multiforme (GBM) is the most prevalent and aggressive primary malignant brain tumor. It diffusely infiltrates the surrounding brain and is characterized by poor prognosis, with a five-year survival rate of 5.5%, despite multimodal therapy (2). Redundant signaling pathways and intratumoral heterogeneity contribute to the inability of conventional and targeted therapies to achieve remission (3–5). Whereas intratumoral heterogeneity traditionally was thought to arise from mutations accumulating and resulting in distinct genotypes, non-genetic heterogeneity from variations in regulatory mechanisms also plays a vital role. Of these regulatory mechanisms, the field of epigenetic modifications as they contribute to the development and progression of GBM has exploded, as shown by this Special Collection.

Epigenetics studies mitotically heritable and stable changes in gene expression resulting from DNA replication and transcription alterations rather than DNA sequence polymorphisms (mutations). Three epigenetic mechanisms have been identified: DNA methylation, histone modification, and noncoding RNA (ncRNA) associated gene silencing (6). DNA methylation involves adding a methyl group directly to a cytosine nucleotide followed by a guanine nucleotide. CpG is shorthand for 5’-C-phosphate-G-3′, where the cytosine and guanine are separated by only one phosphate group, distinguishing this single-stranded linear sequencing from the C-G base pairing for double-stranded sequences. CpGs are often surrounded by other CpGs forming a CpG Island (CGI). CpGs are commonly located in promoter regions because their methylation reduces the interaction between DNA and transcription factors (6). Histone modifications include acetylation, methylation, phosphorylation, and ubiquitylation of histone proteins. Histone modifications alter nucleosome DNA-histone interactions and can facilitate or prevent transcription (6). Noncoding RNAs (ncRNAs) have function despite not being translated into proteins. They participate in DNA methylation, histone modification, and direct gene silencing. Small ncRNAs consist of <200 nucleotides (nt) and include microRNAs (miRNA; 17–23 nt) and small interfering RNAs (siRNA; 20–30 nt). Long non-coding RNAs (lncRNAs) consist of >200 nt and include linear and circular RNAs (7).

**Figure 1:**
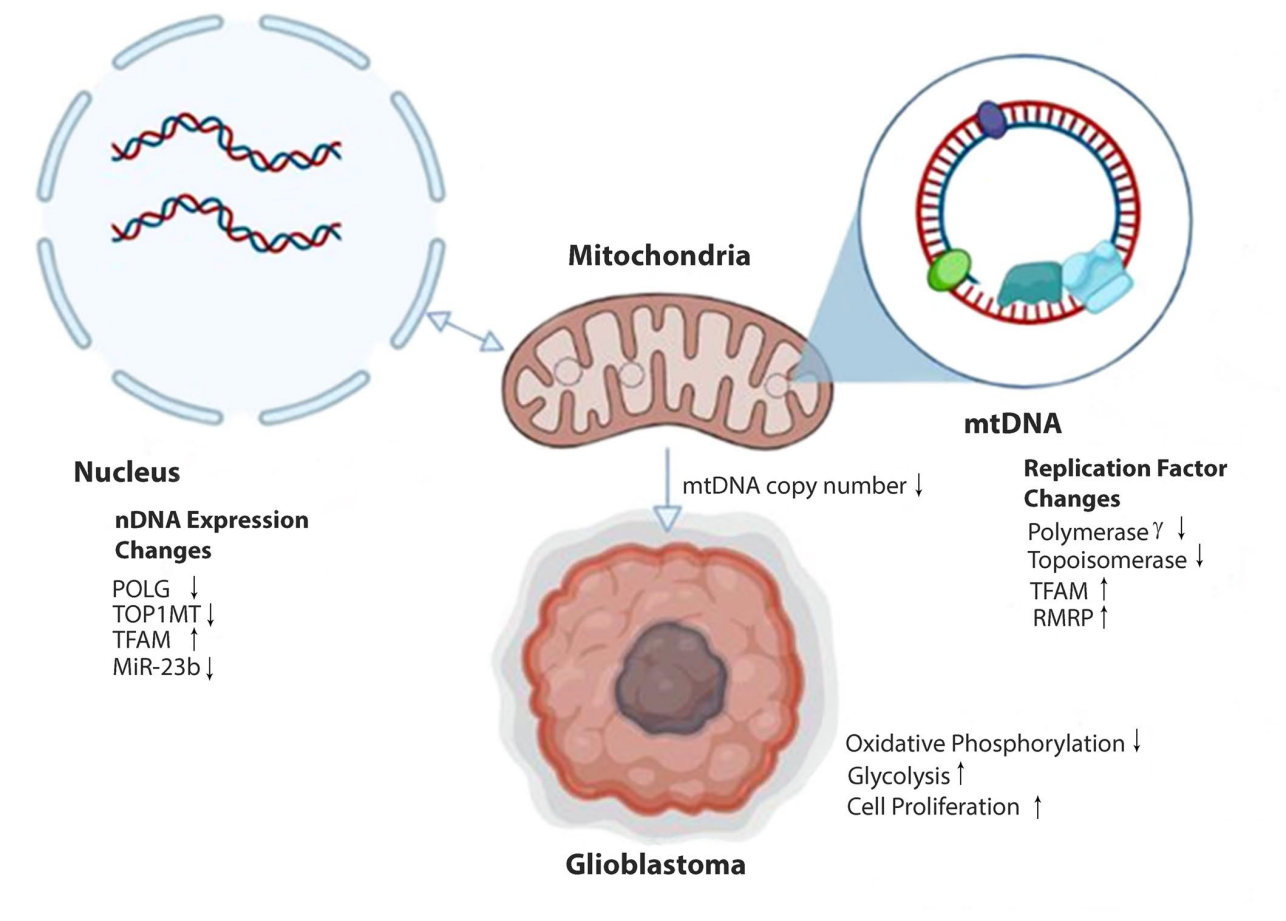
Epigenetic alterations affecting mitochondrial DNA expression in glioblastoma. Methylation of nDNA genes POLG and TOPIMT reduces production of Polymerase γ and Topoisomerase proteins and decreases transport and availability in the mitochondria (anterograde signaling). MiR-23b transcription is reduced in gliomas, increasing the expression of TFAM within the nucleus. Transport of IncRNA RMRP to the mitochondria is increased. Reduced Polymerase γ and Topoisomerase and elevated TFAM and RMRP alter mtDNA copy number and gene expression. Decreases in mtDNA copy number can signal epigenetic modifications of nDNA within the cell nucleus (retrograde signaling). Ultimately, these epigenetic modifications result in reduced oxidative phosphorylation capabilities in the mitochondria. In response, the stem cell adopts glycolytic metabolism and a proliferative glioblastoma phenotype.

Malignant tumors, including gliomas, favor abnormal energy production via aerobic glycolysis and show inherent resistance to apoptosis (8–10). Mitochondrial dysfunction could contribute to GBM pathophysiology by altering metabolic pathways and energy production, but these mechanisms are poorly understood. Mitochondria have a genome (mitogenome) to direct their function and communication with the nuclear genome (11). Recently, it has been shown that mitochondrial DNA (mtDNA), like nuclear DNA (nDNA), is regulated by epigenetic mechanisms through a process referred to as mitoepigenetics (Figure 1) (12).

Traditionally, mtDNA consists of 37 genes: 13 encoding polypeptide components of the respiratory chain, 22 tRNAs, and two rRNAs. mtDNA also contains a significant noncoding region termed the displacement loop (13). However, evidence is emerging that the mitochondrial transcriptome also includes mitochondrial-derived ncRNAs (14).

The mitochondrial proteome consists of approximately 1,500 proteins. nDNA encodes most respiratory chain components and proteins required for the synthesis, expression, and regulation of mitochondrial genes. The nuclear coding of most mitochondrial proteins explains why interest in GBM epigenetics has focused on nDNA rather than mtDNA (15). However, given the association between GBM pathogenesis and mitochondrial dysfunction, the epigenetic regulation of the small 16.6 kb mtDNA genome may be an untapped study area of incredible value. This review discusses the complexity of mitochondrial epigenetics, highlighting the roles mtDNA methylation, mtDNA packing, and ncRNA may play in GBM pathogenesis. We will describe epigenetic changes involving mitochondria that could enhance GBM pathogenicity. These include epigenetic changes to mtDNA directly altering mtDNA expression and epigenetic modifications of nDNA indirectly affecting mtDNA expression.

## 2. Mitochondrial epigenetics

Mitochondrial epigenetics is a largely unexplored field, but the literature is rapidly growing as our understanding of mtDNA and the mitochondrial proteome deepens. The three most common types of nDNA epigenetic modification can be applied with some modification to mtDNA. Increasing evidence shows mtDNA methylation is associated with mtDNA transcriptional regulation and copy number (16, 17). Unlike nDNA, mtDNA lacks histones. mtDNA is packaged instead into nucleoid structures, in which mtDNA lies in the center surrounded by peripheral proteins (18). In 2011, scientists discovered ncRNAs inside mitochondria and created the first comprehensive map of the human mitochondrial transcriptome (14). We will review the three categories of epigenetic alterations in mtDNA (methylation, modification of nucleoid proteins, and ncRNA) in the context of glioma pathogenesis and potential therapeutic targets.

## 3. DNA methylation of mitochondrial DNA

Methylation of the nuclear genome plays a critical role in mitochondrial function through its effect on mtDNA replication and copy number. Human embryonic and neural stem cells have extensive nDNA methylation in the early stages of development but become demethylated as cells differentiate and increase their mtDNA copy numbers (19, 20). An adequate copy number of mtDNA is essential to produce the machinery needed to engage in oxidative phosphorylation and meet ATP demands (21–23). The genome of glioblastoma cells stays heavily methylated. Many studies have shown that the mtDNA copy number is lower in glioma cells than in healthy cells (24–27). Low mtDNA copy number appears to lead glioma cells to rely on glycolysis to produce ATP instead of oxidative phosphorylation, promoting cell proliferation (28). Therefore, methylation of the nuclear and mitochondrial genomes of glioblastoma cells has been studied to understand how this epigenetic mechanism impacts mtDNA replication and mtDNA copy number (Table 1) (23, 28–30).

**Table 1:** Epigenetic modifications of DNA as therapeutic targets for potential drugs/treatments in gliomas.

Epigenetic modification type	Therapeutic target	Drug/Treatment
Methylation of DNA	Nucleus: POLG and TOP1MT Mitochondria: mtDNA	Vitamin C and 5-Azacytidine
Mitochondrial nucleoid modifications	Nucleus: TFAM	KLF16 and Melatonin
Noncoding RNA	Nucleus: RMRP and TFAM	siRNAs and miR-23b

### 3.1. 5-Azacytidine reduces the efficacy of DMNT1 and TOP1MT and increases mtDNA copy number in glioma cell lines

MtDNA copy number is associated with the methylation of exon 2 of DNA polymerase gamma, POLG (28, 31). POLG, found on chromosome 15q25, encodes the mitochondrial-specific DNA polymerase, Pol γ, which is essential in mtDNA replication (32, 33). In the HSR-GBM1 model, exon 2 of POLG is highly methylated. However, using 5-azacytidine (5-Aza) resulted in significant mtDNA copy number increases in the HSR-GBM model (28). 5-Aza irreversibly binds the methylating enzyme DNA methyltransferase 1 (DMT1), reducing its ability to methylate DNA. Therefore, 5-Aza treatment reduced methylation of exon 2 of POLG, increased POLG expression, and increased mtDNA copy number (34). The use of demethylating agent 5-Aza also appeared to induce long-term differentiation of these cells (34).

The TOP1MT gene encodes for the mitochondrial-specific topoisomerase, which facilitates replication and transcription of mtDNA by relieving tension and supercoiling (35). Exon 8 and intron 9 of the TOP1MT gene were heavily methylated in the HSR-GBM1 model. However, TOP1MT expression and mtDNA copy number increased in response to the 5-Aza treatment (23). This further suggests that the demethylation of mtDNA replication factors leads to an increased mtDNA copy number.

### 3.2. Vitamin C enhances TET1 and increases mtDNA copy number in glioma cell lines but has unclear benefits in clinical application

Vitamin C enhances ten-eleven translocation methylcytosine dioxygenase (TET1) activity, which demethylates the 5^th^ position of the pyrimidine ring of cytosine on POLG. Like 5-Aza treatments, VitC demethylation of POLG increased POLG expression and mtDNA copy number. HSR-GBM1 cells given VitC treatment did not differentiate fully (36). These studies demonstrated VitC and 5-Aza as potential treatments, although inhibition of DNMT with 5-Aza may be more effective than Vitamin C in decreasing methylation and upregulating mtDNA copy number. 5- Aza and Vitamin C are DNA demethylation agents that promote cell differentiation and may affect various cancers (34, 37–39). Few studies have examined these agents’ anti-glioma effects. One case study demonstrated intravenous vitamin C treatment benefiting a patient with GBM, suggesting the broader applicability of Vitamin C for glioma therapy (40). However, vitamin C enhanced glioblastoma invasiveness. Vitamin C deficiency reduced glioblastoma proliferation *in vitro* (41, 42). 5-Aza’s had better outcomes than VitC in the HSR-GBM1 model, supporting its testing in other models and potential in clinical trials.

### 3.3. Global demethylation of nDNA increases mtDNA copy number in glioma cell lines

Global demethylation of the HSR-GBM1 nuclear genome modified tumor-specific genes and critical mtDNA transcription and replication factors beyond POLG and TOP1MT (23). Demethylation downregulated a subunit of the SEC61 translocon complex (SEC61G) and upregulated the PR domain containing 16 (PRDM16) and serine/threonine-protein kinase (WNK2). DNA demethylating treatments affected cell death, growth, and differentiation pathways (43–45). Demethylation treatment upregulated epidermal growth factor 2 (EGFR2), B-Cell lymphoma 2 (BCL2), telomerase reverse transcriptase (TERT), and MYC. These tumor markers interact with mtDNA transcription and replication factors. Demethylation regulates cellular fate, cellular differentiation, and mtDNA replication (23, 46–49).

### 3.4. Crosstalk of nDNA and mtDNA and methylation of mtDNA in glioma cell lines

Evidence suggests that nuclear and mitochondrial genomic modifications contribute to glioma tumorigenesis (29). When mtDNA levels were decreased to different extents in HSR-GBM1 cells, cells experienced nDNA methylation changes to restore mtDNA copy numbers (29). This further exemplifies the importance of considering nDNA’s role in mtDNA replication when developing therapies targeting mtDNA copy number.

The methylation of mtDNA has not been studied extensively. Scientists debated for years on whether mtDNA could be methylated (50, 51). However, multiple studies have since confirmed mtDNA methylated sites (23, 52–54). Furthermore, mtDNA methylation contributed to mtDNA copy number and tumorigenesis in GBM. Sun et al. used naïve osteosarcoma cells with their original mtDNA (143B^143B^), and mtDNA depleted 143B cell lines repopulated with donor mtDNA from HSR-GBM1 cells (143B^GBM^) and human neural stem cells (143B^NSC^) (30). Each cell population was analyzed at an early and late growth stage. Cell lines with mtDNA derived from hNSCs (143B^NSC^) had higher levels of mtDNA methylation than cell lines with mtDNA derived from GBM or 143B (143B^GBM^ or 143B^143B^) cell lines, suggesting decreased mtDNA methylation is initially necessary to enforce a tumor phenotype (30). The mtDNA copy number in 143B^GBM^ and 143B^143B^ cell lines also increased significantly from the early to late stages, suggesting that increased mtDNA copy number encouraged early tumorigenesis (30, 55). However, although mtDNA methylation decreases and mtDNA copy number increases as tumors first progress, after tumorigenesis is started by sufficient mtDNA copy number, methylation of mtDNA increases. This late event in tumorigenesis restricts mtDNA replication and maintains mtDNA copy numbers in GBM cell lines lower than in non-tumorigenic cell lines (30). This indicates that the mtDNA methylation changes that result in abnormal energy production in GBM occur in later stages of tumorigenesis.

These results give insight into mtDNA’s role in tumorigenesis. They suggest that mtDNA demethylation contributes to increases in mtDNA copy number, which could support demethylation therapy for GBM. The demethylating agents, 5Aza and VitC, significantly reduced mtDNA methylation and could prove helpful as therapies (23). Additional studies of demethylating agents will show if they can change nDNA and mtDNA epigenetics and tumor pathogenesis in glioma and glioblastoma.

## 4. The nucleoid: mitochondrial DNA packaging and expression

Mitochondrial DNA (mtDNA) has no associated histone protein like nDNA The mitochondria’s nucleoid contains mtDNA centrally surrounded by core and peripheral proteins referred to as core and peripheral nucleoid factors (18). Core factors are crosslinked to mtDNA and include (1) transcription factor A, mitochondrial (TFAM), (2) mitochondrial single-strand binding protein (mtSSB), (3) DNA polymerase subunit gamma (POLG), (4) mtRNA polymerase (POLRMT), (5) Lon protease, and (6) DNA helicase Twinkle (56). Initially, the roles of these factors in transcription, translation, and cell-wide signaling were shown. More recently, it has become clear that several package proteins epigenetically modulate mtDNA, like histones modulate nDNA (57).

### 4.1. TFAM is upregulated in GBM

Mitochondrial transcription factor A (TFAM) is a 24-kDa protein encoded by a nuclear gene on chromosome 10. It was initially identified as a transcription factor for mtDNA (58) but is now thought dispensable for mtDNA transcription *in vitro* but crucial for packaging mtDNA within the mitochondria (59). TFAM has two high-mobility group (HMG) domains. These are DNA binding motifs that, upon binding, induce a U-shape confirmation in mtDNA (60, 61). U-turn bending recruits mitochondrial RNA polymerase to the mitochondrial light strand promoter (LSP) site. It is thought that the degree of bending may affect transcriptional activation efficacy (62). The mammalian mitochondria contain about 1 TFAM protein per 15–18 base pairs of mtDNA, making it abundant enough to coat the entire mitochondrial genome (63).

Lee et al. explored the association of TFAM with GBM and if TFAM antagonism could be an anti-GBM therapeutic strategy (64). In their study, a Western blot analysis with an anti-TFAM antibody showed markedly increased protein expression of TFAM in GBM cell lines, especially U343-MG and U373-MG cells. Quantitative real-time PCR (qRT-PCR) showed elevated mRNA levels of TFAM in the U251-MG, U343-MG, and U373-MG GBM cell lines. Human GBM tissues were also stained with anti-TFAM antibodies. Tumor tissue had considerably more TFAM staining than surrounding tissue. The differential expression of TFAM in the REMBRANDT cohort was analyzed and found to have significantly higher TFAM gene transcript levels in GBM, astrocytoma, and oligodendroglioma than normal controls (*p* < 0.0001) (64).

Correia et al. investigated transcript levels of TFAM as they related to GBM overall survival time (65). Using QT-PCR to quantify expression levels of TFAM, they compared TFAM expression of non-neoplastic brain tissue to two GBM subgroups: survival time under 12 months and survival time over 24 months. Although both GBM subgroups had significantly higher levels of TFAM than non-neoplastic brain tissue, the TFAM expression was higher in the 24-month survivors than the 12-month survivors (65).

Pediatric high-grade gliomas (pHGG) are the deadliest childhood CNS cancers. They are characterized by K27M mutations in histones H3.1 and H3.3 and G34R mutations in H3.3 (H3.3G34R) (66–68). H3.3G34R mutations are almost exclusive to hemispheric pHGG and occur in adolescents and young adults (69). Siddaway et al. unexpectedly discovered H3.3G34R localizes to the mitochondria at a higher rate than wild-type H3.3 (70). Furthermore, TFAM associated exclusively with the H3.3G34R mutated histone and not the wild-type histone. The authors hypothesized that H3.3G34R had a metabolic effect on the pHGG cells. They generated a metabolomic profile of H3.3 wild type versus H3.3G34R and showed enriched TCA cycle metabolites and a higher level of mitochondrial metabolism in these cells (70). H3.3G34R may be a useful epigenetic marker for TFAM in pHGG.

Although these studies elucidate an association between gliomas and TFAM, the mechanism underlying TFAM’s contribution to glioma pathogenesis is poorly understood. Levels of TFAM expression in GBM patient specimens did not correlate with GBM survival. Levels of TFAM and mtDNA transcription efficacy may have a complex, non-linear relationship: as TFAM increases, mtDNA assumes a more favorable mtDNA conformation for mtDNA transcription. However, a specific point may exist when TFAM coats mtDNA, reducing transcription efficacy. The best stoichiometry between TFAM and template DNA has not been established in GBM.

### 4.2. TFAM as a therapeutic target

Nonetheless, TFAM may be a strong candidate target for glioma therapeutic interventions. Chen et al. investigated the relationship between Kruppel-like factor (KLF) 16 and TFAM in glioma cell proliferation (71). KLF members are zinc finger-containing transcription factors that regulate oncogenic or tumor-suppressive genes by binding GC-rich DNA sequences in gene promoter regions (72). The expression of KLF16 was found to be robustly reduced in six glioma cell lines and glioma tissues via Western blot studies and real-time PCR analysis. Furthermore, survival analysis showed glioma patients with low KLF16 had a poor prognosis (HR = 2.328, 95% CI = 1.387–4.017, *p* < 0.01). Given these results, KLF16 was postulated to have a tumor-suppressive role in glioma progression. Real-time PCR indicated TFAM expression was downregulated in KLF16-elevated cells and upregulated in KLF16-silenced cells. Using a chromatin immunoprecipitation (ChIP) assay, they showed a high binding affinity of endogenous KLF16 to the GC-rich basic transcriptional element in the TFAM nDNA promoter, indicating KLF16 directly repressed TFAM expression and could serve as a potential therapeutic target (71).

The effects of melatonin (N-acetyl-5-methoxytryptamine), a hormone synthesized from serotonin, on mitochondria have been widely explored, but little is known about how melatonin affects mtDNA and TFAM expression. Franco et al. investigated the relationship between TFAM and melatonin using the GBM cell line U87MG (73). When melatonin was incubated with these cells, the mRNA expression of TFAM and protein levels decreased. The reduction of TFAM resulted in reduced gene expression of mitochondrial NADH dehydrogenase 1, elevated reactive oxygen species (ROS) production, and decreased cell viability. Lastly, they showed that melatonin acts synergistically with temozolomide (TMZ). Cell viability was reduced by 34% by 3 mM melatonin and by 45% by TMZ. Combined treatment of melatonin and TMZ reduced viability by 87% (73).

Additionally, the exact epigenetic mechanism that results in upregulation of TFAM in GBM is unknown. Characterizing this mechanism may be beneficial as it could provide us with targets to reduce TFAM expression in GBM to restore a normal phenotype. One possible target could be nuclear respiratory factor 1, which has been found to suppress the TFAM promoter when methylated *in vitro* (74). However, future studies must look at this effect in GBM models before a therapy can be developed.

These studies demonstrate TFAM plays an integral role in the structure of mtDNA and transcriptional regulation and may serve as a novel epigenetic target for glioma therapy.

## 5. Noncoding RNAs in mitochondria

Noncoding RNAs (ncRNAs) have regulatory and structural functions, not protein template activity. They regulate gene expression by adjusting RNA processing and mRNA stability, modification, and translation (75). NcRNAs represent 70% of the nuclear genome in humans and the third category of epigenetic processes contributing to glioma development and progression (76). Advances in deep sequencing have revealed that the mitochondrial transcriptome results from a complex regulatory, expression, and processing network. ncRNAs participate in mitochondrial gene regulation (14). Two types of ncRNAs have been found inside mitochondria. The first is nuclear-encoded ncRNAs (nuclear-ncRNAs) involved in directional signaling from the nucleus to the mitochondria (anterograde signaling). The second type is the mitochondria-encoded ncRNAs (mt-ncRNAs). The study of mt-ncRNAs is groundbreaking because, until recently, the mitogenome was thought only to include genes encoding polypeptides, tRNAs, and rRNAs.

Several studies mapped many long noncoding RNAs and small noncoding RNAs to the mitochondrial genome. Mercer et al. identified 31 novel miRNAs expressed from 17 distinct loci. The majority (84%) were derived from tRNA genes (14). Rackham et al. identified three lncRNAs with sequences uniquely aligned to the mitochondrial genes encoding ND5, ND6, and Cyt b (77). Interestingly, ND6 is the least abundant mitochondrial encoded protein, perhaps because lncND6 downregulates its expression (77).

Conversely, nuclear-encoded RNAs have been found within the mitochondria. They are thought to regulate the mitochondrial genome by associating with Argonaute (AGO) proteins, forming the RNA-induced silencing complex (RISC) core, and exerting RNA interference (78). Thirteen nuclear miRNAs were found within the mitochondria associating with AGO and mitochondrial mRNA, implying RNAi may regulate mitochondrial biogenesis and function (78). Analysis of the mitochondrial transcriptome showed a nuclear-lncRNA part of the mitochondrial RNA processing endoribonuclease (14). Termed RMRP, this RNA part is important for mtDNA replication and RNA processing. RMRP helps endonuclease cleave mitochondrial RNA at a priming site of mtDNA replication (79). Given the growing cohort of ncRNAs and their epigenetic influence on mitochondrial expression, several studies have postulated this mechanism may contribute to glioma pathogenesis and can be targeted with new therapeutic agents.

### 5.1. RMRP contributes to glioma progression and TMZ resistance

The LncRNA RNA component of mitochondrial RNA processing (RMRP) was first found to promote carcinogenesis in gastric cancer. RMRP expression was recently investigated in low-grade (grade I-II) to high-grade (grade III-IV) glioma cell lines and tissues (80). Glioma tissues expressed significantly more RMRP than normal brain tissues in qRT-PCR experiments (80). Furthermore, lncRNA RMRP upregulation is significantly associated with advanced tumor grade and low Karnofsky Performance Score (KPS), indicating RMRP up-regulation may be involved in glioma progression. Knockdown of RMRP significantly decreased the proliferation of glioma cell lines *in vitro*. These findings suggest that reducing the expression of lncRNA RMRP impairs the transcription of mtDNA and inhibits malignant phenotypes of glioma cells (80).

Liu et al. performed experiments on lncRNAs, including RMRP, to decide if they were involved in regulating the TMZ resistance in gliomas (81). The top 100 upregulated lncRNAs in glioma tumor tissues were identified, including RMRP. RMRP expression levels were higher in tumors isolated from patients with relapsing glioma after TMZ treatment than tumors from TMZ-naïve patients. Liu et al. investigated the role of RMRP in TMZ resistance. Three siRNAs were synthesized for RMRP knockdown. RMRP knockdown increased the cell apoptosis rate 2.6-fold. RMRP depletion weakened the TMZ resistance of TMZ-resistant glioma cell lines and TMZ-treated glioma xenograft tumors (81). These studies demonstrate RMRP antagonism as a potential strategy for increasing the therapeutic efficacy of TMZ against glioblastoma.

### 5.2. TFAM is directly regulated by mIR-23b in glioma

As previously mentioned, mitochondrial transcription factor A (TFAM) is a core protein within the mitochondrial nucleoid structure responsible for creating favorable conformations of mtDNA and increasing transcription efficacy (62). TFAM can act alone as an epigenetic mechanism in glioma pathogenesis. Its susceptibility to miRNA regulation creates an added layer of mitoepigenetic control. MiR-23b is a miRNA highly expressed in several cancers and associated with tumorigenesis (82). Its role in gliomas was investigated using glioma cell lines and tissue specimens. MiR-23b expression levels measured by real-time RT-PCR were significantly lower in glioma than in normal brain tissue. Like other TFAM studies, this study showed that TFAM expression was significantly increased in glioma tissues and positively correlated to the malignancy grade. Cell lines overexpressing TFAM demonstrated increased proliferation and invasiveness. The 3′ untranslated region of TFAM was found to be a direct target of mIR-23b. Cell lines overexpressing miR-23b had decreased proliferation and invasiveness (80). These results suggest TFAM may be a direct target of epigenetic control via miR-23b (Table 1).

### 5.3. Future clinical development of mitochondrial biomarkers and mitochondria-targeted glioma therapeutics

Mitochondrial deregulation is a GBM marker (83). Sixty percent of solid tumor patients have detectable mtDNA mutations within their body fluid cell-free DNA (84, 85). Epigenetic modifications of nDNA and mtDNA contribute to the malignant features and treatment resistance of glioma patients with nDNA and mtDNA mutations. Researchers are testing anti-glioma therapeutic strategies targeting epigenetic modifications in tumor cells and animal models. Treatments demethylating the POLG, TOP1MT, and TFAM genes attempt to increase tumor cell mtDNA copy number, differentiation, and apoptosis (83). Potential therapeutic agents targeting the POLG, TOP1MT, and TFAM genes include Vitamin C, 5-azacytidine, and melatonin. Another epigenetic therapeutic target is lncRNA RMRP expression. Reducing lncRNA RMRP expression levels in glioma restored the sensitivity of tumor cells to TMZ (Table 1). Inhibiting mtDNA transcription may also decrease glioma cell proliferation and invasion (80).

Glioblastoma and other malignant tumors manifest metabolic reprogramming called the Warburg effect, in which cellular glucose uptake is increased, and the glucose metabolite pyruvate is metabolized anaerobically to lactate. This effect occurs even in cancer cells with functional mitochondria under normoxic conditions (86). Glioblastoma also aerobically metabolizes glucose-derived pyruvate and fatty acids in the mitochondria in actively proliferating, high-oxygen-consuming tumor cells (87, 88). Damaged mitochondria accumulate in tumor cells due to impaired mitophagy and produce reactive oxygen species (ROS) that damage and mutate genomic and mtDNA and enhance genomic instability and oncogenesis (89). Glioblastoma combination therapies can be designed to include agents antagonizing the tumorigenic effects of the Warburg effect and mitochondrial genetic and epigenetic aberrations, like tumor proliferation, invasion, free radical production, impaired mitophagy, and reduced apoptosis (90).

## 6. Conclusion

Mitochondria participate in many biological processes, including metabolism, apoptosis, and cellular proliferation. It has been well-reported that the tumorigenicity of malignant tumors, including gliomas, is related to abnormal energy production and inherent resistance to apoptosis. Understanding mtDNA’s contribution to these processes goes beyond the proteins the mitochondrial genome expresses. We are beginning to appreciate how epigenetic mechanisms regulate mtDNA expression and contribute to tumor pathogenicity (Figure 1). Further understanding of mtDNA methylation, alterations in nucleoid packaging of mtDNA, and regulation of mtDNA by noncoding RNAs in glioma cell lines and tissue samples will uncover novel mechanisms underlying glioma progression that may be amenable to targeted therapies.

## Author contributions

CG: conceptualization, writing original draft, review and editing, and visualization. LW: writing, review and editing, and visualization. JH: conceptualization, writing, review and editing, and supervision. All authors contributed to the article and approved the submitted version.

## Funding

This research was funded by the Intramural Research Program of the National Institute of Neurological Diseases and Stroke at the National Institutes of Health, Project Number 1ZIA NS003052-15.

## Conflict of interest

The authors declare that the research was conducted in the absence of any commercial or financial relationships that could be construed as a potential conflict of interest.

## Publisher’s note

All claims expressed in this article are solely those of the authors and do not necessarily represent those of their affiliated organizations, or those of the publisher, the editors and the reviewers. Any product that may be evaluated in this article, or claim that may be made by its manufacturer, is not guaranteed or endorsed by the publisher.
